# Influence of Waxy (High Amylopectin) and High Protein Digestibility Traits in Sorghum on Injera Sourdough-Type Flatbread Sensory Characteristics

**DOI:** 10.3390/foods9121749

**Published:** 2020-11-26

**Authors:** Abadi G. Mezgebe, John R. N. Taylor, Henriëtte L. de Kock

**Affiliations:** 1 Department of Consumer and Food Sciences, University of Pretoria, Private Bag X20, Hatfield, Pretoria 0028, South Africa; abadigebre84@gmail.com (A.G.M.); john.taylor@up.ac.za (J.R.N.T.); 2 School of Nutrition, Food Science and Technology, College of Agriculture, Hawassa University, P.O. Box 05, Hawassa, Ethiopia

**Keywords:** amylopectin, descriptive sensory profiling, flatbread, injera, protein digestibility sorghum, sourdough, teff

## Abstract

Injera, an East African leavened sourdough fermented pancake has remarkable textural properties despite being made from non-wheat flours. However, teff flour, which produces the best quality injera, is expensive and limited in availability. The effects of waxy (high amylopectin) and high protein digestibility (HD) traits in sorghum on injera quality were studied. Eight white tan-plant sorghum lines expressing these traits in various combinations and three normal sorghum types were studied, with teff flour as reference. Descriptive sensory profiling of fresh and stored injera revealed that injera from waxy sorghums were softer, spongier, more flexible and rollable compared to injera from normal sorghum and much closer in these important textural attributes to teff injera. Instrumental texture analysis of injera similarly showed that waxy sorghum injera had lower stress and higher strain than injera from normal sorghum. The improved injera textural quality was probably due to the slower retrogradation and better water-holding of amylopectin starch. The HD trait, however, did not clearly affect injera quality, probably because the lines had only moderately higher protein digestibility. In conclusion, waxy sorghum flour has considerable potential for the production of gluten-free sourdough fermented flatbread-type products with good textural functionality.

## 1. Introduction

Injera, a sourdough fermented leavened pancake-type flatbread, which is a staple food in the East African countries of Ethiopia and Eritrea, is a truly remarkable bread. Injera has a soft aerated cellular texture despite the fact that it is made with non-wheat cereal flours [1]. It is also elastic and highly flexible to the extent that pieces can be tightly rolled. Injera sourdough breadmaking technology has the potential to be the foundation for developing high functional quality flatbread-type bakery products from non-wheat cereals without having to include additives to provide gluten-type, viscoelastic, and gas-holding functionality. It can be assumed that during sourdough fermentation, microflora modify flour starch, protein, and non-starch polysaccharides to help provide these functionalities [2], although this has not been specifically studied.

The nature of the cereal flour also plays an important role in injera quality. The best injera is produced using teff flour. This seems to be due to teff’s unusual starch, which comprises compound granules made up of tiny polygonal granules that have high proportion of amorphous regions [3]. However, teff is a particularly expensive cereal due to its low yield and because its tiny grain size makes mechanized harvesting challenging [4].

Sorghum, which is cultivated in tropical and subtropical areas across the world, is more available and less expensive than teff, due to its wider environmental adaptation and relatively high productivity [5]. Thus, producing injera and other flatbread-type bakery products from sorghum is attractive. However, the poorer texture and more rapid staling of sorghum injera [6] have been identified as major constraints preventing this development. Staling involves physico-chemical changes and consequent effects on sensory attributes such as firming of product texture, increased crumb opacity, and a decline in attractive flavors [7], among these firming is probably the most important. Starch retrogradation is a major cause of firming [8]. Starch retrogradation takes place when disaggregated starch components (amylose and amylopectin chains) in cooked, gelatinized starch realign themselves as the cooked starch cools [9].

Amylopectin, the branched chain-type starch molecule, is less susceptible to reassociation during retrogradation than amylose, the essentially straight chained starch [10]. Amylopectin retrogradation occurs very slowly [11], resulting in slower staling and softer baked products [7]. Inclusion of waxy wheat flour, in place of normal wheat flour, similarly, has been found to exhibit an anti-staling effect and produce softer breads [12,13,14]. In addition, partial substitution with waxy (high amylopectin) corn and potato starch (up to 10–15%) for normal corn/potato mix in gluten-free bread formulations has decreased bread hardness and produced softer breads [15]. The same study found that waxy starch (up to 10%) was an effective factor for reducing the staling of starch and firmness of gluten-free bread, during three days of storage. Furthermore, wheat tortilla (unfermented flatbread) made from waxy wheat flour was found to have greater extensibility after at least three days storage [16].

Texas A&M University has developed, through conventional breeding, novel sorghum lines that express waxy and high protein digestibility (HD) traits [17]. Sorghum lines expressing both the waxy and HD trait have been found to have better flour properties for making dough-based food products [18]. Moreover, sorghum lines with just the HD trait, which results from modified expression of the endosperm kafirin storage proteins, have been shown to have improved composite wheat-sorghum flour dough and bread quality [19].

Thus, with the objective of searching for alternative flour for making injera, improving the quality of sorghum injera and more generally that of sorghum sourdough-fermented flatbread-type bakery products, this work applied descriptive sensory profiling and instrumental texture analysis to evaluate the effects of the waxy and HD traits in sorghum on the sensory attributes of fresh and stored injera as compared with teff injera.

## 2. Materials and Methods

### 2.1. Materials

Eight novel sorghum lines, variously expressing the waxy or HD traits, plus three normal types were studied. They were produced at the Ukulima Research Farm, Limpopo Province, South Africa, in a controlled field trial. The novel sorghum lines were non-tannin white tan-plant types and derived from crosses between lines RTx2907 and P850029 by Texas A&M Agrilife Research, College Station, TX, USA. The RTx2907 line is a waxy and normal protein digestibility line released by Texas A&M Agrilife Research [20], while the P850029 line is a high protein digestibility line that was developed by Purdue University from the high lysine line P721Q [21]. The novel sorghum lines comprised three waxy-normal protein digestibility (WND), one heterowaxy-normal protein digestibility (hWND), two waxy-HD (WHD), one non-waxy-HD (NWHD) and one non-waxy-normal digestibility (NWND) type, as described [22]. The normal sorghum types were white non-tannin sorghum (wNTS) (cultivar PAN 606), red non-tannin sorghum (RNTS) (PAN 8816), and red tannin sorghum (RTS) (PAN 8625). Wholegrain white teff flour (PANNAR Seed variety) obtained from Bloemfontein Teff growers (Bloemfontein, South Africa) was included as a reference.

### 2.2. Milling

The sorghum grains were tempered to 16% moisture, then milled using a twin break roller-type mill (Maximill, Kroonstad, South Africa) to achieve an 84–86% flour extraction rate. The milled grain was separated into four different particle size fractions on vibrating screens of mesh opening sizes 1000, 850, and 710 μm. The overs fractions from the 850 and 710 μm screens were mixed, and re-milled, and combined with the 710 μm through fraction to obtain the desired extraction rate. Then, these were milled using a hammer mill fitted with a 500 μm mesh opening size screen to obtain flours of the required particle size. The flours were stored in tightly closed plastic drums at 8 °C.

### 2.3. Preparation of Injera by the Conventional (Full-Scale) Method

Injera was prepared as described [6], modified to a 1 kg flour sample size by increasing the volumes in proportion. The sorghum flour or teff flour (1 kg) was mixed with 900 mL tap water and hand kneaded for 5 min. Sourdough starter culture (10% of the dough by weight), from a previously fermented injera dough, was added on the top of the dough and mixed. Then, the mixture was allowed to ferment for 3 days at ambient temperature (20 °C). Then, 20% of the fermented dough was mixed with 130 mL water and cooked in 550 mL boiling water (for teff only 67% of these amounts were used) for 2–3 min, cooled to ±45 °C, and added back to the fermenting dough and mixed well. Water (300 mL) was added, and then the batter was allowed to ferment again for 2–3 h, at ambient temperature until it formed a foam of bubbles. The fermented batter (500 mL) was poured in a circular manner onto a 50 cm diameter electrically heated, hot clay griddle (mitad) (heated for 25 min to reach an injera undersurface temperature of about 200 °C). The injera was baked covered for about 2 min until cooked through. Then, the injera was removed by lifting it off the hot griddle with the aid of a circular woven grass mat. At least three injera from each sorghum line and teff were prepared.

### 2.4. Analyses

#### 2.4.1. Chemical Composition

Flour moisture, protein, fat, and ash were determined using AACC Methods 44-15A, 46-30, 30-25, and 08-17, respectively [23]. Starch content was determined using the Megazyme total starch assay procedure (zmyloglucosidase/α-amylase method) (Megazyme International, Bray, Ireland). All chemical analyses were repeated at least three times.

#### 2.4.2. Descriptive Sensory Analysis

Descriptive sensory analysis of fresh and stored (2 and 4 days at 5 °C) injera was conducted using a 10-member trained sensory panel. The panelists gave their informed written consent having first been informed in writing of the nature of the injera samples and the purpose of the study. Ethical approval for the sensory study was obtained from the University of Pretoria Faculty of Natural and Agricultural Sciences Research Ethics Committee (study approval number EC180417-186). Sensory profiling of the products was performed using the generic descriptive analysis method [24].

The panel was trained in two sessions of 2 h per day during which descriptions for aroma, appearance, texture, flavor, and aftertaste sensory properties of injera with definitions, reference standards, and methodology of evaluation were developed (Table 1). The attributes were evaluated using an 11-point line scale (0–10) anchored with verbal descriptions at extreme ends.

Evaluation of the fresh and stored injera was performed in duplicate during sessions of 2 h per day. The product evaluation was done in a sensory laboratory with individual booths following standard good sensory practices [25]. Rolled triangular pieces of injera (a 1/16th segment) were presented to the panelists in small round glass bowls on white trays at ambient temperature. Fresh injera was evaluated within 3 h after baking. All samples were presented with randomly selected three-digit codes. Drinking water was provided for rinsing between samples. Responses were recorded by the panelists on computer using Compusense^®^ Five release 4.6 (Compusense, Guelph, Canada).

#### 2.4.3. Instrumental Texture Analysis

Textural properties (stress and strain) of injera were determined using a 3-point bending rig with an aluminum bar (5 mm wide and 90 mm long) mounted on a SHIMADZU EZ-L texture analyzer (Kyoto, Japan). The injera were cut into uniform strips (6 × 3 cm) using a sharp knife. Single injera strips were sealed in zip-lock-type polyethylene bags and stored at 5 °C for 0, 2, and 4 days at 5 °C. The thickness of the fresh and stored injera strips was measured using calipers. The testing profile was as follows: pre-test speed (1.0 mm/s), test speed (3.0 mm/s), post-test speed (10.0 mm/s), distance (15 mm), and trigger type (0.049 N, Auto). The injera strips were placed over the vertical struts (30 mm apart) of the bending rig and clamped in place at both ends. The strips were compressed at a constant rate of 3.3 mm/s over a distance of 15 mm. The peak force (N) of 6 strips from each injera was measured and stress and strain (%) at the maximum elastic extensibility were calculated according to [26], as follows:

Stress (kPa) σ = 3FL/(2bd^2^), (1)

Strain % ε = ∆L/L × 100,(2)

where F is the force (N); L is the support span length; b is the width; d is the thickness; ΔL is the change in L (calculated by subtracting the initial L from compressed L (mm)).

#### 2.4.4. Statistical Analyses

The effect of sorghum type and teff on the chemical composition of the flour was analyzed through one-way ANOVA using Statistica version 8 software, StatSoft, Inc. (Tulsa, Oklahoma, OK, USA). The instrumental texture data, to determine the effect of sorghum type on injera, were analyzed separately for 0, 2, and 4 days of storage using one-way ANOVA. A three-way ANOVA (flour type, storage time, and panelist) with flour type X storage time interaction was applied to the descriptive sensory data. Principal component analysis (PCA) of the sensory data (product categorization option in Sensory data analysis Y = P(products) + J(judges) + S(sessions), the analysis includes attributes by discriminating power at *p*-value < 0.05) and correlation analysis of sensory and instrumental texture data were performed using XLSTAT version 2016.03 (Addinsoft, New York, NY, USA).

## 3. Results and Discussion

### 3.1. Chemical Composition of the Flours

The sorghum lines exhibited a wide range of starch amylopectin contents (Table 2). The five waxy sorghum lines (WND1, WND2, WND3, WHD1, and WHD2) had starch amylopectin contents, ranging between 87.9 and 94.1%. All the other six sorghums had lower starch amylopectin contents (75.6–85.4%), indicating that they were non-waxy and heterowaxy sorghum types [10]. The starch amylopectin of the teff variety was 77.6%, showing that it was non-waxy. There was no significant difference in starch content (*p* ≥ 0.05) between the flours of the 11 sorghum lines, nor did they differ from the teff. The starch content of the novel sorghum line flours was, however, slightly higher than their grains (73.0–78.5%), as reported by [22]. This is because of bran removal when milling to the 84–86% extraction rate as starch is located in the starchy endosperm. The protein content of the sorghum flours varied somewhat, from 11.2 to 13.5%, within the range of their grains (12.0–13.8%) [22] and of waxy, heterowaxy, and HD sorghum genotypes studied by [27], but generally slightly lower than the teff flour (13.4%). The fat content of the sorghum flours varied quite widely, between 2.6 and 4.6% and was generally higher than the teff, 2.9%, but within the range of the sorghum genotypes [27]. The ash content of the sorghum flours (1.3–2.2%) was much lower than the teff, because of removal of the mineral-rich bran. As all novel sorghum lines and normal sorghum types were similar in starch and protein contents, the lines were considered to be suitable to study the influences of the waxy and HD traits on their injera making quality.

### 3.2. Descriptive Sensory Analysis of Injera

The trained sensory panel generated 29 injera quality descriptors (Table 1). These were in the following six sensory categories: aroma, visual appearance, texture as measured by hand touch and in mouth, flavor, and aftertaste. For each category, there were four to seven attributes. The lexicon included 13 attributes previously used by [6] and 16 additionally defined attributes. The additional attributes were developed by the sensory panel in this study considering that the injera made from teff is strongly preferred, as explained above. For this reason, the focus of the discussion is on differences in the attributes as compared with teff injera.

Table 3 presents a summary of the main effects (flour type, storage time, panelist) and of interaction between flour type x storage time on the sensory properties of the injera. The type of flour used and storage time both had significant effects (*p* < 0.05) on the majority of the sensory properties. This indicates that the attributes selected by the panel were relevant for the objectives of the study, i.e., to determine whether sorghum lines with the novel waxy or HD traits affected injera quality. With the exception of dry mouthfeel, all the additional attributes developed by the panel in this present work exhibited significant flour type or storage time effects. This indicates the complexity of describing the sensory characteristics of injera and the requirement for a large number of attributes. A significant panelist effect was noted for all sensory attributes. This implies that the individual panelists may have used different parts of scales to indicate their responses. Panelists were trained to quantify relative differences in sensory properties among samples and were not calibrated to use absolute values on ratings scales. A significant panelist effect is a common occurrence in sensory analysis studies as a result of differences in physiological sensitivities of panelists, sample order influences, etc. [29]. The trained descriptive sensory panel can be viewed as an instrument with 10 specific sensors providing a holistic view of the product sensory properties. The significant panelist effect does not negate the findings with respect to the observed effects of flour type and storage time.

The interaction of flour type x storage time was significant for two injera appearance properties (shininess of the top surface and perceived eye evenness) and five texture properties assessed by hand. This indicates that injera made with the different types of flours were behaving differently during storage. These effects were investigated by subjecting the sensory data to PCA.

**Figure 1:**
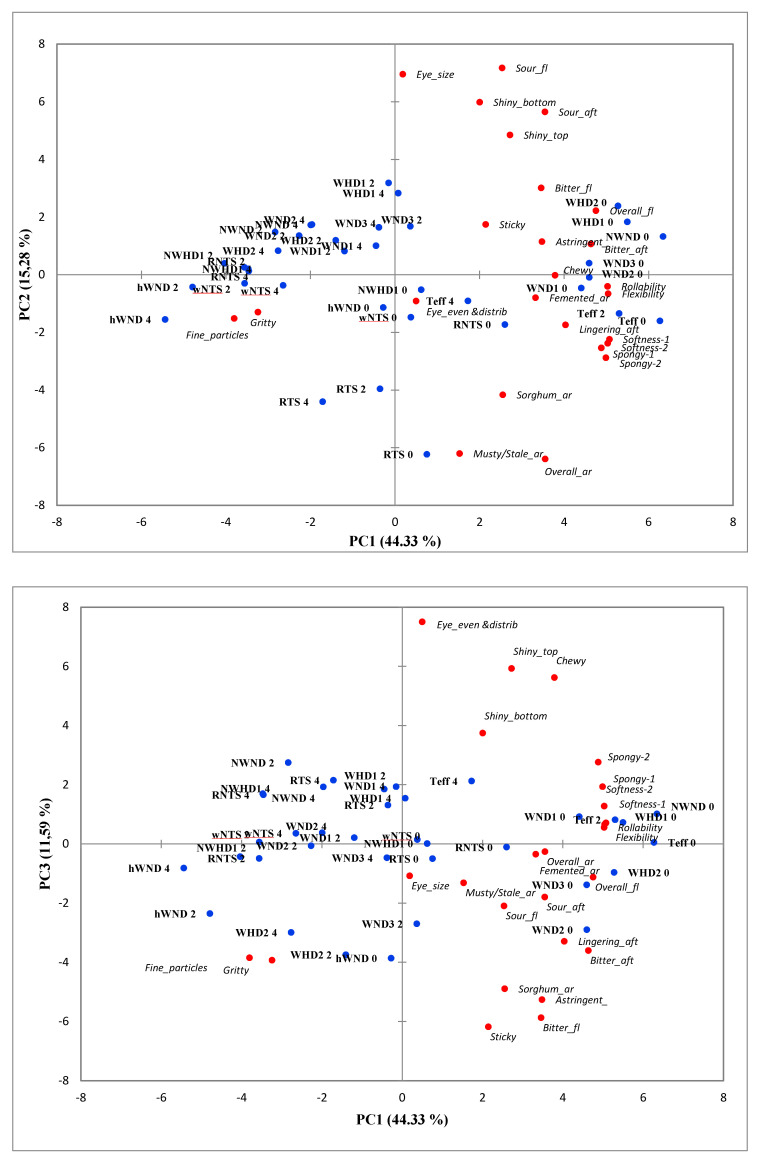
Principal component analysis (PCA) biplots (PC1, PC2, and PC3) of novel sorghum lines, normal sorghum types, and teff and their injera quality attributes of freshly prepared injera (0), and injera stored for 2 days (2) and 4 days (4) at 5 °C. NWND, non-waxy-normal protein digestibility; NWHD, non-waxy-high protein digestibility; hWND, heterowaxy-normal protein digestibility; WND1, WND2, and WND3, waxy-normal protein digestibility lines 1, 2, and 3; WHD1 and WHD2, waxy-high protein digestibility lines 1 and 2; wNTS, white non-tannin sorghum; RNTS, red non-tannin sorghum; RTS, red tannin sorghum; ar, aroma; fl, flavor; aft, aftertaste.

With PCA of the means for attributes of the different injera treatments (significant for both flour type and storage time) (Figure 1), PC1 (44% of the variance) explained the texture related properties of the injera, with samples that were more rollable, flexible, softer, and spongier located on the right of the plot. PC1 additionally separated injera with more intense bitter and lingering aftertaste, astringent fermented aroma, and bitter flavor also on the right of the plot. PC2 explained 15% of the variance and separated samples to the top part of the plot with larger eyes, sour flavor, and aftertaste from those in the bottom part of the plot that displayed mustier stale aroma, sorghum aroma, and overall aroma, which were particularly associated with injera made from the red tannin sorghum (RTS). PC3 added 12% to the explanation of the variance separating stickier, more bitter and astringent injera to the bottom of the plot from those with evenly distributed eyes, shiny tops, and chewy bite being at the top of the plot. The gradual move with storage time to the left of the plots for all injera flour types is indicative of textural changes caused by staling as they became less soft, spongy, flexible, and rollable.

Concerning the effect of sorghum type, with fresh injera, the five waxy sorghum lines were closest in the desirable textural characteristics of softness, sponginess, flexibility, and rollability to teff injera. With storage, however, injera made from all the various sorghum types were characterized by more fine gritty particles as compared with teff injera. In general, the aroma and flavor intensities of injera from all the flour types decreased with storage time. With the exception of sourness, injera flavor and aftertaste were not greatly affected by flour type (Table 3 and Figure 1). This is probably because these attributes are mainly a consequence of the microorganisms responsible for fermentation and as the same starter culture was used for all injera fermentation, this would account for the lack of effect. The highly significant effect (*p* < 0.001) of flour type on sour flavor and aftertaste (Table 3) was possibly because the flours contained differing levels of fermentable sugars. The finding of the current study is consistent with [6] that found white pericarp sorghum cultivars characterized by a sourer flavor and aftertaste as compared with other sorghum cultivars due to rapid lactic acid fermentation.

**Figure 2:**
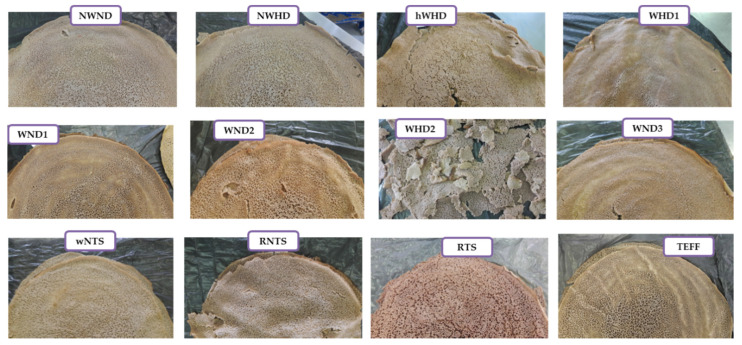
Visual appearance of the fresh sorghum and teff injera. NWND, non-waxy-normal protein digestibility; NWHD, non-waxy-high protein digestibility; hWND, heterowaxy-normal protein digestibility; WND1, WND2, and WND3, waxy-normal protein digestibility lines 1, 2, and 3; WHD1 and WHD2, waxy-high protein digestibility lines 1 and 2; wNTS, white non-tannin sorghum; RNTS, red non-tannin sorghum; RTS, red tannin sorghum.

With the exception of the RTS sorghum, the visual appearance of the injera in terms of shininess and eye size, eye evenness, and distribution, was not clearly related to the different flour types (Figure 1 and Figure 2), despite the highly significant correlations (*p* < 0.05) with the attributes (Table 3). This is presumably because these characteristics were consequences of the sourdough fermentation and baking conditions, as well as flour starch and protein properties. The RTS sorghum injera had large and coarse “eyes” (the pores resulting from the gas cells formed in the batter during baking, setting into a sponge-type cellular structure). Binding of the sorghum tannins with starch and protein was probably responsible for the different eye patterns of the RTS injera. The color of the injera from the different flour types ranged from off-white, through tan to reddish brown (Figure 2). This observation is consistent with [6] where it was reported that the range of colors of injera from different sorghum cultivars was due to differences in pericarp color, glume color, and tannin content, and also with [30] who observed that tannin level affected sorghum injera color.

**Figure 3:**
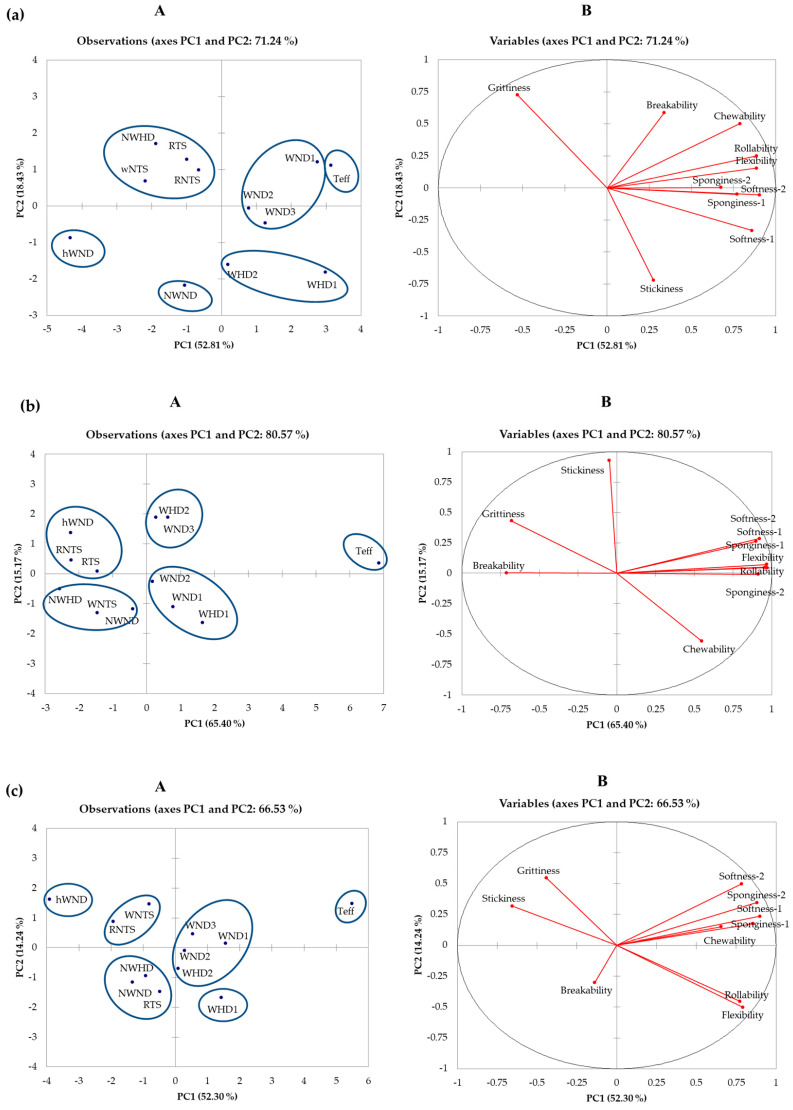
PCA of novel sorghum lines, normal sorghum types, and teff and their injera textural quality attributes of (**a**) freshly prepared injera, (**b**) stored injera (2 days at 5 °C), (**c**) stored injera (4 days at 5 °C); (**A**) Sorghum line PC scores. NWND, non-waxy-normal protein digestibility; NWHD, non-waxy-high protein digestibility; hWND, heterowaxy-normal protein digestibility; WND1, WND2, and WND3, waxy-normal protein digestibility lines 1, 2, and 3; WHD1 and WHD2, waxy-high protein digestibility lines 1 and 2; wNTS, white non-tannin sorghum; RNTS, red non-tannin sorghum; RTS, red tannin sorghum; (**B**) Textural attribute PC loadings. Softness-1 (by hand touch), softness-2 (in-mouth sensation), flexibility, rollability, stickiness, sponginess-1 (by hand), sponginess-2 (in-mouth), chewiness, breakability, and grittiness.

Because of the poor texture of sorghum injera, particularly stored injera, it is the most important sensorial difference between sorghum and teff [6]. Hence, the PCA was applied to the texture related sensory attributes separately for the fresh, two, and four days stored injera made with the different flours (Figure 3), considering there were significant effects (*p* < 0.05) on the particular attributes (Table 3). With the fresh injera (Figure 3a), all five waxy sorghum lines, both those with and without the HD trait, were similar in texture to teff injera with respect to the desirable in-hand and in-mouth attributes of softness, sponginess, flexibility, and rollability (right hand side of the plot). The two waxy-HD lines were, however, also associated with the less desirable attribute of sticky injera as measured by hand. In contrast, all the normal starch and the heterowaxy starch sorghum lines were on the left side of the plot and were more associated with in-mouth grittiness (small particle), a less desirable texture.

After storage for both two days and four days at 5 °C, injera from all five waxy sorghums (WHD1, WND1, WND2, WHD2, and WND3) remained more similar in texture to teff injera than the non-waxy and heterowaxy lines sorghum lines (NWND, NWHD, wNTS, RNTS, RTS, and hWND) (Figure 3b,c). However, with time, there was a progressively increasing separation of the waxy sorghum injera from the teff injera, with teff injera remaining more closely associated with the desirable textural attributes of rollability and flexibility. Nevertheless, it is clear from the descriptive sensory panel data that the waxy (high amylopectin) trait in sorghum was highly associated with the most important desirable injera textural attributes of softness, sponginess, flexibility, and rollability for both stored as well as fresh injera. 

The improved texture of injera made from waxy sorghum (WHD1, WND1, WND2, WHD2, and WND3) was probably a consequence of the lower susceptibility of amylopectin to reassociation during retrogradation as compared with normal starch [10], and its better water-holding property as compared with amylose [7], which results in much slower starch retrogradation [11], producing slower staling and softer baked products [7]. The findings of this current study are in agreement with research on pan-type breads from different cereals where waxy barley starch [31], waxy wheat flour inclusion [12,13,14], and pregelatinized waxy rice flour addition [32] were found to produce softer breads and retard their staling. They are also in agreement with the observation of [33] of softer crumb texture in French bread when using wheat flours of low starch amylose content (15.4 to 16.6%). In addition, with regard to gluten-free breads, the findings are consistent with those of [15] of softer textured bread made with starch where a regular maize/potato starch mix was partially replaced with waxy maize/waxy potato starch mix.

Concerning the HD trait, it did not have a beneficial effect on sorghum injera texture (Figure 1 and Figure 3). This is probably because the increased level of protein digestibility of the cooked flours of the three HD lines was relatively small, 6–20% [22]. The apparent stickiness of fresh injera of the two waxy lines that also expressed the HD trait (Figure 3a) may be due to the floury endosperm texture of sorghum lines when combining the waxy and HD traits [18]. The floury endosperm of the high protein digestibility sorghums may have resulted from the reduction in β-kafirin, which is hydrophobic [34]. The reduction in β-kafirin would increase the hydrophilicity of sorghum protein, which could result in greater water holding for a longer time and delay the hardening of the gel [18].

### 3.3. Instrumental Texture Analysis of Injera

To corroborate the descriptive sensory injera textural data, the instrumental texture of the same injera as were evaluated by the sensory panel were measured in terms of maximum stress (firmness) and strain (extensibility) (Table 4). Stress of the sorghum injera increased considerably during storage at 5 °C, from 124–159 kPa (fresh) to 157–226 kPa (two days stored) and 201–386 kPa (four days stored), as did the teff injera. Fresh injera from four of the five waxy lines (WND1–3 and WHD2) had similar stress to teff and RTS injera (*p* ≥ 0.05). The injera of these waxy lines had significantly lower stress as compared with injera from the non-waxy lines (NWND and NWHD) and normal sorghum types (wNTS and RNTS) (*p* < 0.05). However, injera from the other waxy line WHD1 and the heterowaxy line hWND had similar stress (*p* ≥ 0.05) to injera from the non-waxy sorghums. This is probably due to the fact that these two lines were relatively lower in starch amylopectin (87.9% and 85.4%, respectively) as compared with the other four waxy lines (88.7–94.1%) (Table 2). After storage, injera from the waxy lines WND1–3 and WND3 had significantly higher stress than teff injera (*p* < 0.05), but lower than NWND, NWHD, wNTS, and RNTS injera at two days storage and lower than these lines and RTS sorghum at four days storage. The generally lower stress of fresh and stored injera from the waxy lines as compared with the normal sorghums is indicative of the waxy trait resulting in softer injera that staled less severely than that of the normal sorghums, as described by the sensory panel (Figure 3). The instrumental texture results are in general agreement with a study by [35] which showed that bread produced with waxy wheat flour blended with normal wheat flour was softer and staling was retarded. As stated, the positive effects on injera softness and staling are probably a consequence of the slow retrogradation and good water holding of amylopectin.

Strain of the sorghum injera decreased considerably during storage, from 28.0–40.7% (fresh) to 10.1–17.7% (two days stored) and 4.3–7.8% (four days stored), as did the teff injera (Table 4). Fresh injera of all five waxy sorghum lines, WND1–3 and WHD1 and -2, had similar strain to teff injera (*p* ≥ 0.05). These injera also had significantly higher strain (*p* < 0.05) as compared with injera from all the sorghum lines. However, stored injera from the five waxy lines had significantly lower strain (*p* < 0.05) as compared with stored teff injera, but they had significantly higher strain (*p* < 0.05) as compared with stored injera from all the other sorghum types. The higher strain of fresh and stored injera of waxy lines as compared with injera made from non-waxy and even heterowaxy sorghum flour is indicative of the high amylopectin trait resulting in more flexible and less brittle injera (Figure 3).

A correlation matrix of the sorghum injera textural properties measured by instrumental texture analysis with those measured by the sensory panel showed that the stress of both the fresh and stored injera (Table 5) were significantly negatively correlated (*p* < 0.05) with both softness-1 (hand tactile feel) and softness-2 (in mouth sensation) measured by the sensory panel. Furthermore, strain was significantly positively correlated with injera softness-1 and -2 (*p* < 0.01), flexibility (*p* < 0.05), and rollability (*p* < 0.05) measured by the sensory panel. The significant correlations between instrumental stress and strain and the important injera sensory textural attributes of softness, flexibility, and rollability corroborate the descriptive sensory panel data and vice-versa. Furthermore, it indicates that the stress and strain measured by instrumental texture analysis can be used to predict these injera textural attributes in laboratories where there is no trained sensory panel or to minimize the time and cost of analysis. The current findings are in agreement with [36] that reported sensory attributes like hardness and springiness of different foods were both accurately predicted by textural profile analysis via instrumental measurement including strain and stress.

## 4. Conclusions

Sorghum with the waxy (high amylopectin starch) trait produces softer, spongier, more flexible, and rollable injera than normal starch sorghum, with no evident effect on aroma, appearance, flavor and aftertaste quality attributes. When stored, waxy sorghum also produces softer and flexible injera as compared with normal sorghums. The better injera quality from waxy sorghum is probably due to the slower retrogradation and better water holding capacity of amylopectin starch. The high protein digestibility trait, however, did not clearly affect injera quality, probably due to the relatively small improvement in protein digestibility in these lines. White tan-plant waxy sorghum can produce injera of better quality than normal sorghum and much closer to teff injera. Thus, waxy sorghum has considerable potential as a flour for the production of good functional quality additive-free and gluten-free sourdough fermented flatbread and wrap-type bakery products.

## Figures and Tables

**Table 1:** Lexicon used to describe the sensory characteristics of injera made from the various sorghum types and teff.

Sensory Category	Attributes	Definition/Reference	Scale Anchors (0, 10)
Aroma	Fermented	Intensity of aroma associated with beer	No fermented aroma, intense fermented aroma
	Sorghum	Intensity of aroma associated with sorghum porridge	No sorghum aroma, intense sorghum aroma
	Musty	Intensity of aroma associated with moldy smell	No musty aroma, intense musty aroma
	Overall aroma	Overall intensity of aroma of injera	No aroma, intense overall aroma
Appearance	Shininess *	The property of having a smooth shiny/glossy/lustrous surface	Not shiny surface, very shiny surface
	Eye ^#^ size *	The size of pores formed on surface	Very large, very small
	Eye evenness and distribution *	The distribution and evenness of the pores	Not evenly distributed, very evenly distributed
Texture (assessed by hand)	Softness-1 *	The measure of resistance to deformation	Not soft, very soft
	Flexibility	The property of bending easily without breaking	Not flexible, very flexible
	Rollability *	The ease to roll the injera	Not rollable, very rollable
	Stickiness *	Degree to which a product tends to be glutinous	Not sticky, very sticky
	Sponginess-1	Property resembling a sponge; light, porous and compressible	Not spongy, Very spongy
Texture (assessed in mouth)	Chewiness	A measure of the ease to masticate or chew the injera	Not chewy, very chewy
	Softness-2 *	The measure of resistance to deformation	Not soft, very soft
	Sponginess-2	Property resembling a sponge; light, porous and compressible	Not spongy, very spongy
	Breakability	The measure of resistance to breaking	Not breakable, very breakable
	Grittiness *	Degree to which small particles were noticed during mastication.	Not gritty, very gritty
	Dry mouthfeel	Degree to which the injera feels dry while chewing and absorbs saliva	No dry mouthfeel, intense dry mouthfeel
Flavour	Sourness *	Fundamental taste sensation elicited by acids	No sour taste, intense sour taste
	Bitterness *	Fundamental taste sensation elicited by caffeine	No bitter taste, intense bitter taste
	Starchy	Flavour associated with carbohydrate-rich products (bread and pasta)	No starchy flavour, intense starchy flavour
	Sweetness *	Fundamental taste sensation elicited by sugar	No sweet taste, intense sweet taste
	Overall flavour	Overall intensity of flavor of injera	No flavour, intense overall flavour
Aftertaste	Sourness *	Intensity of the sour taste remaining in the mouth after swallowing the injera	No sour aftertaste, intense sour aftertaste
	Bitterness *	Intensity of the bitter taste remaining in the mouth after swallowing the injera	No bitter aftertaste, intense bitter aftertaste
	Dry mouthfeel	Degree to which mouth feels dry after swallowing the injera	No dry mouthfeel, intense dry mouthfeel
	Fine particles	Degree to which small particles remained in the mouth after swallowing.	No fine particles, many fine particles
	Astringent	Chemical sensation associated with puckering of tongue caused by substances such as tannins perceived after swallowing the injera	No astringent taste, intense astringent taste
	Lingering	Length of time which the aftertaste lasts after swallowing	No lingering aftertaste, intense lingering aftertaste

# Eye is the term used by injera consumers to describe the gas cells; * Attributes used by [6], attributes without symbol were developed by the panel in this present work.

**Table 2:** Chemical composition of flours (g/100 g, dry basis) of novel sorghum lines with waxy and high protein digestibility (HD) traits, normal sorghum types, and teff.

Sorghum Line	Starch Amylopectin (%) *	Starch	Protein	Fat	Ash
NWND	79.9 ^a^ ± 1.1	80.3 ^a^ ± 1.2	12.8 ^cd^ ± 0.5	2.7 ^a^ ± 0.0	1.7 ^ab^ ± 0.2
NWHD	81.1 ^ab^ ± 1.0	78.0 ^a^ ± 2.8	12.9 ^cd^ ± 0.0	4.1 ^bcd^ ± 0.7	1.7 ^ab^ ± 0.0
hWND	85.4 ^bc^ ± 0.1	76.9 ^a^ ± 3.0	13.1 ^cd^ ± 0.0	3.7 ^abcd^ ± 0.2	1.4 ^a^ ± 0.1
WHD1	87.9 ^c^ ± 0.7	80.0 ^a^ ± 3.8	11.7 ^a^ ± 0.1	4.1 ^bcd^ ± 0.2	1.5 ^a^ ± 0.0
WND1	88.7 ^cd^ ± 0.9	76.6 ^a^ ± 2.5	13.5 ^d^ ± 0.0	4.6 ^d^ ± 0.4	1.8 ^ab^ ± 0.1
WND2	89.1 ^cde^ ± 2.5	79.3 ^a^ ± 4.0	13.2 ^cd^ ± 0.3	3.0 ^ab^ ± 0.1	1.6 ^ab^ ± 0.1
WHD2	93.6 ^de^ ± 2.0	80.0 ^a^ ± 1.3	12.8 ^cd^ ± 0.0	2.6 ^a^ ± 0.0	2.2 ^b^ ± 0.2
WND3	94.1 ^e^ ± 0.4	80.7 ^a^ ± 1.4	11.9 ^ab^ ± 0.1	3.1 ^ab^ ± 0.0	1.3 ^a^ ± 0.0
wNTS	75.6 ^a^ ± 1.3	81.2 ^a^ ± 1.1	11.4 ^a^ ± 0.2	3.5 ^abcd^ ± 0.1	1.3 ^a^ ± 0.0
RNTS	77.2 ^a^ ± 0.8	79.0 ^a^ ± 1.2	12.7 ^bc^ ± 0.1	4.3 ^cd^ ± 0.4	1.6 ^ab^ ± 0.4
RTS	79.1 ^a^ ± 1.2	78.5 ^a^ ± 3.8	11.2 ^a^ ± 0.2	3.3 ^abc^ ± 0.1	1.3 ^a^ ± 0.1
Teff	77.6 **	76.3 ^a^ ± 0.8	13.4 ^cd^ ± 0.0	2.9 ^a^ ± 0.1	3.3 ^c^ ± 0.2

Values are mean ± standard deviation (*n* = 2). Values in a column with different letters in superscript are significantly different (*p* < 0.05). NWND, non-waxy-normal protein digestibility; NWHD, non-waxy-high protein digestibility; hWND, heterowaxy-normal protein digestibility; WHD, waxy-high protein digestibility; WND, waxy-normal protein digestibility; wNTS, white non-tannin sorghum; RNTS. red non-tannin sorghum; RTS. red tannin sorghum. * Sorghum starch amylopectin data [22] and ** teff starch amylopectin data [28].

**Table 3:** Summary of main and interaction effects on the sensory attributes of injera and their level of statistical significance.

		Flour Type	Storage Time	Panellist	Flour Type × Storage Time
Aroma	Fermented aroma	ns	***	***	ns
	Sorghum aroma	*	***	***	ns
	Musty/stale aroma	***	*	***	ns
	Overall aroma	***	***	***	ns
Appearance	Shininess of top *	***	ns	***	*
	Shininess of bottom *	***	ns	***	ns
	Eye size *	***	ns	***	ns
	Eye evenness *	***	*	***	**
Texture (assessed by hand)	Softness-1 *	***	***	***	***
	Sponginess-1	***	***	***	ns
	Flexibility	***	***	***	*
	Rollability *	***	***	***	**
	Stickiness *	***	***	***	*
Texture (assessed in mouth)	Chewiness	**	***	***	ns
	Softness-2 *	***	***	***	ns
	Sponginess-2	***	***	***	ns
	Breakability	ns	**	***	ns
	Grittiness *	*	ns	***	ns
	Dry mouthfeel	ns	ns	***	ns
Flavour	Sourness *	***	ns	***	ns
	Bitterness *	**	***	***	ns
	Starchy	ns	**	***	ns
	Sweetness *	ns	***	***	ns
	Overall flavor	***	***	***	ns
Aftertaste	Sourness *	***	***	***	ns
	Bitterness *	ns	***	***	ns
	Dry mouthfeel	ns	ns	***	ns
	Fine particles	***	*	***	ns
	Astringent	**	*	***	ns
	Lingering	**	***	***	ns

* Attributes used by [6], attributes without symbol were developed by the panel in this present work, *** *p* < 0.001, ** *p* < 0.01, * *p* < 0.05, ns = not significant.

**Table 4:** Effects of the waxy and high protein digestibility HD traits on instrumental textural properties of fresh and stored sorghum injera as compared with teff injera.

Sorghum Line	Stress (kPa) at Maximum Elastic Extension			Strain (%) at Maximum Elastic Extension		
	Storage Days (5 °C)			Storage Days (5 °C)		
	0	2	4	0	2	4
NWND	159 ^f^ ± 14	217 ^fg^ ± 12	351 ^h^ ± 20	34.2 ^bc^ ± 2.2	10.1 ^a^ ± 0.6	5.6 ^bc^ ± 0.6
NWHD	153 ^def^ ± 10	226 ^g^ ± 16	386 ^i^ ± 20	33.5 ^bc^ ± 2.1	11.6 ^ab^ ± 1.1	4.7 ^ab^ ± 0.5
hWND	150 ^def^ ± 5	188 ^cde^ ± 8	296 ^efg^ ± 18	35.8 ^cd^ ± 2.2	11.0 ^a^ ± 1.0	5.3 ^ab^ ± 0.5
WHD1	152 ^def^ ± 11	202 ^ef^ ± 16	303 ^fg^ ± 10	39.9 ^de^ ± 2.6	14.9 ^cd^ ± 1.2	7.2 ^d^ ± 0.8
WND1	137 ^bc^ ± 9	178 ^bcd^ ± 10	247 ^cd^ ± 19	42.3 ^e^ ± 1.2	14.4 ^c^ ± 1.0	6.8 ^d^ ± 0.5
WND2	130 ^bc^ ± 10	157 ^b^ ± 13	266 ^de^ ± 10	40.7 ^e^ ± 1.7	16.8 ^de^ ± 1.2	6.9 ^d^ ± 0.7
WHD2	126 ^ab^ ± 7	161 ^b^ ± 6	215 ^bc^ ± 15	39.3 ^de^ ± 2.4	17.7 ^e^ ± 0.7	7.8 ^d^ ± 0.5
WND3	124 ^ab^ ± 8	166 ^bc^ ± 12	201 ^ab^ ± 9	39.9 ^de^ ± 3.5	17.1 ^e^ ± 1.4	7.6 ^d^ ± 0.8
wNTS	157 ^ef^ ± 10	204 ^efg^ ± 11	294 ^ef^ ± 20	32.0 ^abc^ ± 2.7	13.3 ^bc^ ± 1.0	4.3 ^a^ ± 0.5
RNTS	146 ^cdef^ ± 6	207 ^efg^ ± 8	330 ^gh^ ± 26	30.6 ^ab^ ± 2.0	11.4 ^ab^ ± 1.0	5.5 ^b^ ± 0.8
RTS	140 ^bcde^ ± 8	197 ^def^ ± 18	278 ^def^ ± 23	28.4 ^a^ ± 1.9	14.3 ^c^ ± 1.2	5.3 ^ab^ ± 0.6
Teff	111 ^a^ ± 6	120 ^a^ ± 8	169 ^a^ ± 12	42.5 ^e^ ± 2.7	21.5 ^f^ ± 1.5	10.6 ^e^ ± 0.5

Values are mean ± standard deviation (*n* = 6). Values in a column with different letters in superscript are significantly different (*p* < 0.05). NWND, non-waxy-normal protein digestibility; NWHD, non-waxy-high protein digestibility; hWND, heterowaxy-normal protein digestibility; WND1, WND2, and WND3, waxy-normal protein digestibility lines 1, 2, and 3; WHD1 and WHD2, waxy-high protein digestibility lines 1 and 2; wNTS, white non-tannin sorghum; RNTS, red non-tannin sorghum; RTS, red tannin sorghum.

**Table 5:** Correlation matrix of textural properties of the freshly prepared and 2- and 4-day stored sorghum injera measured by the descriptive sensory panel and by instrumental texture analysis (stress and strain).

	Soft-1	Flexibility	Rollability	Stickiness	Spongy-1	Chewiness	Soft-2	Breakability	Grittiness	Dry MF	Stress	Strain
Fresh injera												
Flexibility	0.600 *											
Rollability	0.584 *	0.961 **										
Stickiness	0.555 ns	0.106 ns	0.103 ns									
Spongy-1	0.650 *	0.579 *	0.535 ns	0.001 ns								
Chewiness	0.479 ns	0.805 **	0.893 **	−0.070 ns	0.437 ns							
^1^ Soft-2	0.940 **	0.675 *	0.670 *	0.347 ns	0.688 *	0.640 *						
Breakability	0.454 ns	0.305 ns	0.396 ns	−0.034 ns	0.562 ns	0.445 ns	0.599 *					
Grittiness	−0.605 *	−0.479 ns	−0.412 ns	−0.629 *	−0.335 ns	−0.158 ns	−0.386 ns	0.192 ns				
Dry MF	−0.691 *	−0.608 *	−0.509 ns	−0.282 ns	−0.800 **	−0.317 ns	−0.680 *	−0.336 ns	0.365 ns			
Stress	−0.729 **	−0.373 ns	−0.445 ns	−0.505 ns	−0.071 ns	−0.400 ns	−0.690 *	−0.270 ns	0.411 ns	0.121 ns		
Strain	0.725 **	0.618 *	0.633 *	0.456 ns	0.404 ns	0.460 ns	0.714 **	0.141 ns	−0.590 *	−0.417 ns	−0.750 *	
Spongy-2	0.501 ns	0.523 ns	0.494 ns	−0.153 ns	0.895 **	0.405 ns	0.592 *	0.701 *	−0.223 ns	−0.576 *	−0.026 ns	0.273 ns
Two-days stored												
Flexibility	0.791 **											
Rollability	0.741 **	0.987 **										
Stickiness	−0.010 ns	−0.374 ns	−0.376 ns									
Sponginess	0.935 **	0.886 **	0.867 **	−0.230 ns								
Chewiness	0.154 ns	0.302 ns	0.344 ns	−0.482 ns	0.362 ns							
Soft-2	0.946 **	0.813 **	0.770 **	−0.164	0.957 **	0.383 ns						
Breakability	−0.613 *	−0.647 *	−0.690 *	0.070 ns	−0.648 *	−0.091 ns	−0.494 ns					
Grittiness	−0.723 **	−0.639 *	−0.619 *	0.208 ns	−0.699 *	−0.311 ns	−0.706 *	0.663 *				
Dry MF	−0.132 ns	−0.204 ns	−0.263 ns	0.183 ns	−0.172 ns	−0.356 ns	−0.053 ns	0.661 *	0.426 ns			
Stress	−0.718 **	−0.571 ns	−0.483 ns	−0.007 ns	−0.552 ns	0.188 ns	−0.616 *	0.478 ns	0.744 **	0.020 ns		
Strain	0.948 **	0.827 **	0.773 **	−0.123 ns	0.870 **	0.205 ns	0.900 **	−0.570 ns	−0.787 **	−0.168 ns	−0.779 **	
Spongy-2	0.583 *	0.655 *	0.705 *	−0.035 ns	0.636 *	0.367 ns	0.669 *	−0.505 ns	−0.402 ns	−0.157 ns	−0.300 ns	0.579 *
Four-days stored												
Flexibility	0.402 ns											
Rollability	0.348 ns	0.961 **										
Stickiness	−0.262 ns	−0.612 *	−0.548 ns									
Sponginess	0.911 **	0.526 ns	0.514 ns	−0.484 ns								
Chewiness	0.604 *	0.748 **	0.692 *	−0.726 **	0.660 *							
Soft-2	0.950 **	0.386 ns	0.367 ns	−0.282 ns	0.854 **	0.669 *						
Breakability	−0.146 ns	0.112 ns	−0.043 ns	0.107 ns	−0.274 ns	−0.047 ns	−0.236 ns					
Grittiness	−0.732 **	−0.395 ns	−0.354 ns	0.328 ns	−0.721 **	−0.283 ns	−0.604 *	0.144 ns				
Dry MF	−0.289 ns	0.340 ns	0.299 ns	−0.357 ns	−0.108 ns	0.148 ns	−0.289 ns	0.531 ns	0.175 ns			
Stress	−0.733 **	−0.445 ns	−0.518 ns	−0.015 ns	−0.640 *	−0.392 ns	−0.742 **	0.253 ns	0.633 *	0.443 ns		
Strain	0.735 **	0.694 *	0.697 *	−0.401 ns	0.694 *	0.820 **	0.793 **	0.023 ns	−0.423 ns	0.110 ns	−0.694 *	
Spongy-2	0.274 ns	0.256 ns	0.263 ns	−0.162 ns	0.295 ns	0.334 ns	0.376	−0.171 ns	−0.128 ns	0.399 ns	−0.124	0.452 ns

Two tailed correlation significant (indicated in bold) at * *p* < 0.05, ** *p* < 0.01, and *** *p* < 0.001 and ns, not significant. Soft-1, softness measured by hand tactile feel; Soft-2, softness measured in mouth; Spongy-1, sponginess measured by hand tactile feel; Spongy-2, sponginess measured in mouth; Dry MF, dry mouthfeel.
